# Cutaneous melanoma

**DOI:** 10.1054/bjoc.2001.1771

**Published:** 2001-05

**Authors:** S Négrier, B Fervers, C Bailly, V Beckendorf, D Cupissol, J F Doré, T Dorval, J R Garbay, C Vilmer

**Affiliations:** Centre Léon Bérard, Lyon; Cancer Research Campaign, Paris; Centre Alexis Vautrin, Nancy; Centre Val d'Aurelle Paul-Lamarque, Montpellier; Institut Curie, Paris; Centre René Huguenin, Saint-Cloud, France


*British Journal of Cancer* (2001) **84**(Supplement 2), 81–85
© 2001 FNCLCC
doi: 10.1054/ bjoc.2001.1771, available online at http://www.idealibrary.com on IDEAL®

### Cutaneous melanoma

**S Négrier[1], B Fervers[1,2], C Bailly[1], V Beckendorf[3], D Cupissol[4], JF Doré[1], T Dorval[5], JR Garbay[6] and C Vilmer[6]**

[1]Centre Léon Bérard, Lyon; [2]FNCLCC, Paris; [3]Centre Alexis Vautrin, Nancy; [4]Centre Val d'Aurelle Paul-Lamarque, Montpellier; [5]Institut Curie, Paris; [6]Centre René Huguenin, Saint-Cloud, France

Cutaneous melanoma is a highly malignant tumour. The incidence has increased dramatically over the last few decades and is now estimated at between 4–5000 new cases per year in France.

The term 'naevus', unless otherwise specified, refers to an acquired or congenital benign melanocytic tumour (commonly known as a naevus, naevi or mole). A melanoma can develop de novo, or within a pre-existing benign naevus. A melanoma can arise in any area containing melanocytes, but approximately 90% are cutaneous tumours.

These recommendations refer to localized primary tumours, those presenting with regional nodes and those with distant metastases. The management of mucosal, visceral and ophthalmic melanomas is not covered.

These recommendations are based on literature published until the end of 1998. Data published since does not change these recommendations. An update is planned for early 2001.

### RISK FACTORS AND PREVENTION

The identification of risk factors is useful for the prevention of melanoma. Two types of risk factors have been identified: individual (constitutional) factors (photosensitivity, numerous naevi, atypical naevi, giant congenital naevi, personal or family history of melanoma) and behavioural factors (excess exposure to sun or artificial ultraviolet rays) (level of evidence A).

The primary prevention of melanoma depends on a reduction in exposure to ultraviolet rays, either solar or artificial. Secondary prevention is based on the early diagnosis of a melanoma at a curable stage and the surveillance of high-risk patients.

### DIAGNOSIS AND HISTOPATHOLOGICAL ASSESSMENT

Diagnosis is the first step in the management of a melanoma (standard). Certain clinical criteria in a pigmented cutaneous lesion are suggestive of malignancy (standard). The criteria are classified as follows:

- criteria A : asymmetry
- criteria B : irregular borders
- criteria C : heterogeneous colour
- criteria D : large diameter
- criteria E : evolution (recent change) – this criteria must co-exist with at least one of the preceding criteria.

Some authors use the three change criteria: change in size, colour and shape. Other authors have proposed a list of seven criteria: three major criteria (change in size, change in colour, change in shape) and four minor criteria (diameter greater than 7 mm, hypersensitivity, bleeding and inflammation).

Epiluminescence microscopy (ELM), or dermatoscopy, can improve the clinical diagnosis of pigmented lesions. It can differentiate a melanocytic from a non-melanocytic pigmented lesion such as a seborrheic keratosis, pigmented basal-cell carcinoma or haemangioma. Dermatoscopy for the early diagnosis of a melanoma should only be used by those familiar with the technique. Its accuracy depends on the experience of the dermatologist. Currently, it cannot be recommended as a routine technique.

The standard practice for the management of cutaneous melanocytic lesions thought to be malignant is a limited but complete excision under local anaesthetic of the lesion with a narrow rim (2 mm) of normal skin. The incision will usually be elliptical with the long axis parallel to the skin lines to allow for re-excision with minimal skin loss. If the initial excision is incorrect (for example a transverse incision across a forearm rather than a longitudinal incision down the arm) a skin graft may be necessary at the time of re-excision.

Cutaneous lesions should be excised rather than biopsied for the following reasons:

- if the lesion is benign, there is no need for further treatment
- there is a risk of misdiagnosis if a melanocytic lesion is only partially examined
- an examination of the entire lesion is necessary to assess all histological parameters, maximum thickness in particular.

The thickness of the lesion and the clearance of the margins can only be determined by histological examination. Tissue destruction can compromise the final diagnosis and the assessment of histological prognostic factors. A scalpel rather than a laser or electro-coagulation should therefore be used for the excision. All excisional or incisional biopsies must be sent to the pathologist (standard). Frozen sections must be discouraged. Excision margins should be documented in the operation note. Standard histological examination is generally sufficient.

The amount of information that can be obtained from the excised sample depends on the quality of the specimen. The age, the sex of the patient and the site of the lesion are mandatory for the histopathological interpretation. Immunohistochemistry alone cannot provide a diagnosis of malignancy and should not be used routinely. It can, however, confirm the melanocytic nature of a cutaneous lesion with an unusual presentation, whether it is primary or secondary (particularly for non-pigmented lesions) and can improve the detection of occult metastases in lymph nodes.

The histopathological report must include at least (NIH consensus conference of 1992 and the French Consensus Conference of 1995) the:

- diagnosis of the melanocytic nature of the lesion and confirmation of its malignancy

- maximum tumour thickness in millimetres (according to Breslow's method).
- assessment of completeness of excision of invasive and in situ components; microscopic measurement of the shortest extent of clearance
- level of invasion (Clark)
- presence and extent of regression
- presence and extent of ulceration.

Optional parameters are:

- histological type and special variants
- pre-existing lesion
- mitotic rate
- vascular invasion
- neurotropism
- cell type
- tumour lymphocyte infiltration
- growth phase; vertical or radial.

A complete clinical examination is standard for an isolated primary melanoma to detect a second primary melanoma and/or metastases, including nodal metastases. The clinical examination must include an examination of the entire skin surface (including scalp) and of all regional nodes (standard). In the case of an isolated primary melanoma with no clinically detectable nodes, there is no need for further investigations. Studies have shown that the risk of nodal involvement is correlated with the maximum thickness and level of invasion of the primary tumour. There is no indication for routine nodal dissection for diagnosis or staging; this has no impact on survival and the surgery carries significant morbidity. There is no effective adjuvant treatment. An ultrasound of superficial regional nodes is indicated for cases of clinical uncertainty (option).

Sentinel nodes can be identified by blue dye or a radio-labelled colloid marker. This is not a routine procedure, since no gain in survival has been demonstrated after nodal dissection and/or with adjuvant treatment in the case of tumour involvement of a sentinel node. Nodes can be identified electively with a morbidity of almost zero. These techniques facilitate planned lymph-node dissection to detect microscopic metastases.

Technetium lymphoscintigraphy can localize nodal drainage pathways for melanomas situated in medial regions (head, neck, trunk). This is only of interest if prophylactic nodal dissection is planned within a prospective study (e.g. the sentinel node study according to Morton's technique).

In cases of regional or metastatic nodal involvement, a search for other metastatic lesions is justified to guide subsequent therapy. In about 10% of cases distant asymptomatic metastases are discovered at the time of diagnosis of involved nodes. There is no consensus regarding the efficacy of imaging techniques to detect distant metastases; abdominal, thoracic and cerebral CT scans seem the most useful. Other investigations can be made according to physical signs. Immunoscintigraphy is still in the process of development and is not recommended in routine practice to demonstrate clinically undetectable metastases.

For patients with metastatic disease at any site at presentation, a search for the primary tumour must include a careful history of any previous treatment to a cutaneous lesion and an examination of the whole body (standard). In the case of an isolated node, cytological analysis of a sample obtained by fine-needle aspiratation can be considered as a diagnostic, aid but a definitive diagnosis can only be made following histopathological examination of tissue (standard). If a pigmented lesion is found to contain melanin by histochemistry, there is no need for immunohistochemistry. If the lesion is achromic, immunohistochemistry is necessary to confirm melanocytic origin.

Histopathological examination of lymph nodes requires at least one section through the centre of each node (standard). The number of nodes examined, the number of involved nodes and any capsular rupture must be reported (standard). If there is nodal involvement, the number of nodes greater than 3 cm should be defined by TNM classification criteria. If there is no evidence of nodal invasion on microscopy, immunohistochemistry should be used for the detection of occult metastases.

Polymerase chain reaction (PCR) is not a validated technique in melanoma and should not be used routinely.

### CLASSIFICATION AND PROGNOSTIC FACTORS

Five different international classifications are accepted to define the clinical stages of melanoma. The AJCC/UICC classification (option) is used most often in English-speaking countries and in the literature.

For single excisable melanomas, the Breslow index is the most powerful and most commonly used prognostic factor (level of evidence A). There is a close correlation between survival and the Breslow index. For thin melanomas (Breslow index less than 1 mm), the level of invasion (Clark's level) has prognostic value. Other clinical criteria (sex, age, site of the lesion) have prognostic value (level of evidence C), but these parameters have complex inter-relations and their value is low when compared to that of the Breslow index.

When a melanoma presents with locoregional node involvement, the number of involved nodes is the most important prognostic factor (level of evidence B). For patients with metastatic melanoma, the number of metastatic sites and the time interval between the primary tumour and the metastases appear/to be the most important prognostic factors.

### TREATMENT MODALITIES

### Surgery

The standard treatment of a localized primary melanoma is surgery (Figure 1). After initial surgery, a wider second excision may be

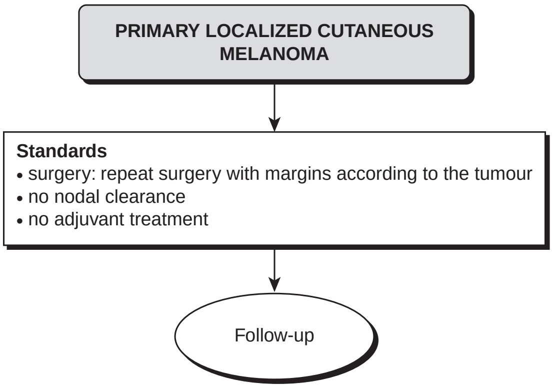

**Figure 1** Treatment of primary localized cutaneous melanoma

 *© 2001 FNCLCC*

necessary with margins depending on the results of the initial histological examination (standard). These margins will vary according to the depth of the tumour, specifically the Breslow index (standard). In France there are two guidelines which are used to base the extent of excision margins; those established by ANDEM in 1994 and those of the French consensus conference in 1995. The ANDEM system is recommended on the basis of simplicity and compatability with published data (level of evidence B).

The required margin of excision is dependent on the thickness of the lesion:

- melanoma in situ (non invasive): margin of 0.5 cm from edge to edge
- Breslow melanoma ≤ 1 mm: margin of 1 cm
- melanoma with Breslow ≥ 1 mm, ≤ 2 mm: margins of 1–2 cm
- melanoma with Breslow ≥ 2 mm, ≤ 4 mm: margins of 2 cm
- melanoma with Breslow > 4 mm: margins of 3 cm.

For melanomas of Breslow thickness less than 1 mm, the same recommendations were made at the time of the NIH consensus conference in 1992. There is no consensus dealing with the excision margins in cases where tumour regression is noted on histological examination. The excision margins should be those for lesions in the category immediately above the actual thickness.

The value of regional nodal dissection is controversial and the data in the literature are contradictory. The theoretical aims of prophylactic nodal dissection are to improve overal survival (by diminishing the risk of metastases by spread from involved nodes) and to provide prognostic information (although this will not be useful until there is effective adjuvant treatment). Nodal dissection should only be considered for lesions on the limbs where there is a single route of lymph drainage. Surgery is always associated with some morbidity, especially in the lower limbs. The early complication rate is between 10–15% in the best series of inguinal dissection. The rate of late lymphoedema is between 6–15% in the lower limbs and 6% in the upper limbs. At the present time, routine nodal dissection after excision of an isolated cutaneous melanoma is not recommended (level of evidence C).

### Radiotherapy

There is no indication for radiotherapy in operable melanoma excised with adequate margins (standard) (Figure 1).

### Adjuvant therapy

There is no survival advantage when adjuvant chemotherapy is given after excision of an isolated tumour or after excision of nodal metastases (level of evidence B). Adjuvant chemotherapy should not be given outside a trial. No survival advantage has been shown from the use of hormone therapy (progestogens) in the adjuvant setting (level of evidence B). Adjuvant hormone therapy should not be used outside a study. The results of controlled studies of immunotherapy in the adjuvant setting have not been consistent, but suggest that a benefit may be possible for certain sub-groups of patients (level of evidence C). Adjuvant intra-arterial infusions have not been shown to be of definite benefit (level of evidence B). Intra-arterial perfusion therapy with hyperthermia and/or chemotherapy and/or immunotherapy should not be considered as primary therapy and must only be undertaken by experienced teams within controlled studies.

### THERAPEUTIC STRATEGY

### Treatment of localized primary melanoma

The standard treatment of a localized primary melanoma is surgery (Figure 1). After initial surgery, a wider second excision may be necessary with margins depending on the results of the initial histological examination (standard). These margins will vary according to the depth of the tumour, specifically the Breslow index (standard). There is no indication for radiotherapy (standard) or adjuvant chemotherapy (level of incidence B, standard).

### Treatment of regional node involvement

The standard treatment of patients presenting with regional node involvement, whether it be the presenting feature, a synchronous presentation with the primary tumour, or the first indication of recurrence, is surgical dissection of involved nodes (Figure 2). No other treatment has been shown to be superior (level of evidence B). The appropriate surgery is nodal dissection, although there is no consensus regarding its extent. After complete nodal dissection, there is no indication for any further treatment (level of evidence B). Adjuvant radiotherapy has not been shown to be of benefit after complete nodal clearance (level of evidence C). Radiotherapy is an option in the case of incomplete nodal clearance, for example in the case of fixed nodes, extensive invasion or capsular disruption.

Controlled studies of adjuvant immunotherapy have given inconsistent results but suggest that there may be a benefit in subgroups of patients, in particular those patients with metastatic regional nodes (level of evidence C). Adjuvant therapy with interferon has shown a significant advantage in terms of overall survival in one study (level of evidence C), which was not confirmed by a subsequent study. This therapy has considerable toxicity and patients must be strictly selected and closely followed during treatment. Adjuvant immunotherapy is an option for operable patients with regional nodal metastases, but cannot be recommended

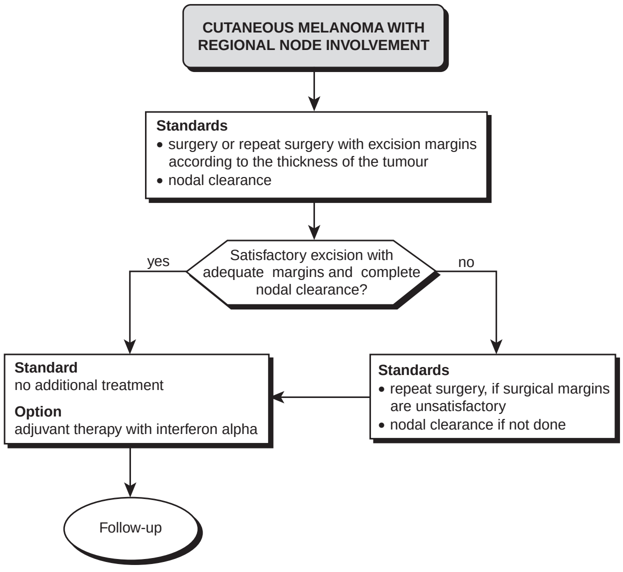

**Figure 2** Treatment of cutaneous melanoma with regional node involvement

© 2001 FNCLCC

as primary/first-line treatment in operable melanoma with or without involved nodes. Further randomized controlled studies are required to confirm the potential role of adjuvant immunotherapy.

### Treatment of an isolated local recurrence

Surgical excision is the standard treatment.

### Treatment of metastatic melanoma

There is no standard therapy for metastatic melanoma (Figure 3). With the possible exception of the resection of in-transit metastases or slowly developing single metastases, there is no curative treatment. Therapy should be adapted according to the number of lesions, the rate of progression of the disease and the performance status of the patient. Some palliative chemotherapy and immunotherapy protocols have resulted in significant tumour regression with a median duration of remission of 4 to 5 months. Conventional palliative chemotherapy is dacarbazine. Polychemotherapy has not been shown to be superior to dacarbazine alone with respect to survival (level of evidence B). The exact role of immunotherapy in the treatment of metastatic disease remains to be determined.

### Treatment of a Hutchinson's melanotic freckle or lentigo maligna (melanoma in situ)

The standard treatment is surgical excision with a margin of 0.5 cm (see Figure 4).

### Treatment of in-transit metastases

The standard treatment for in-transit metastases (more than 3–5 cm from the primary lesion) is surgical excision (Figure 5). In the case of very numerous metastases, treatment by perfusion of an isolated limb can be considered (level of evidence C). This should only be done by a specialized team trained in this technique.

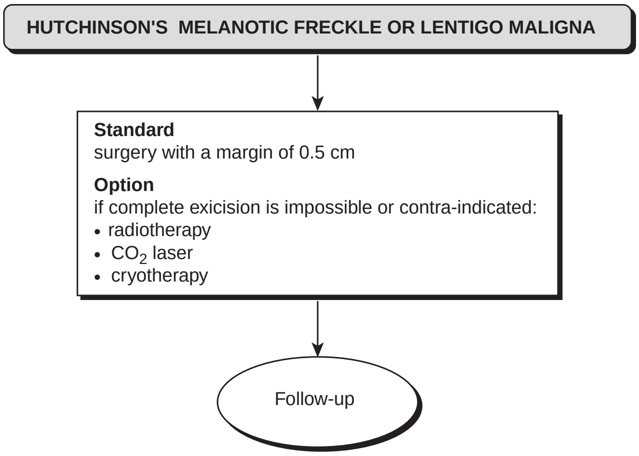

**Figure 4** Treatment of Hutchinsons melanotic freckle

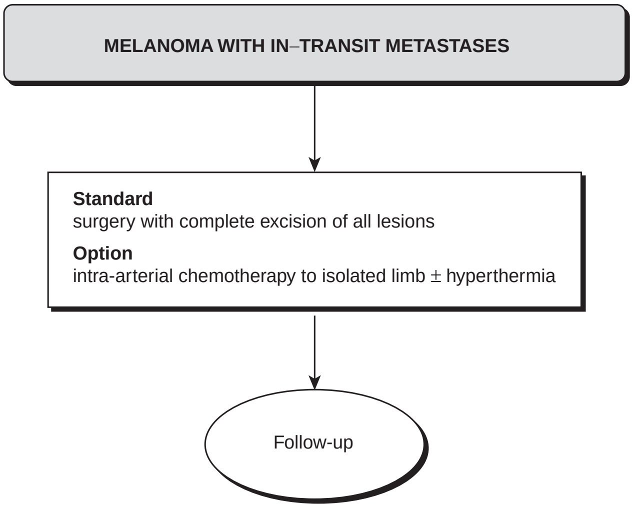

**Figure 5** Treatment of melanoma with in-transit metastases

### FOLLOW-UP

Follow-up is based on clinical examination (standard). Self-surveillance should be encouraged by the provision of relevant patient information (standard). Surveillance is indicated in all cases throughout life (standard), especially in the case of an isolated operable melanoma. There is no case for routine blood tests or imaging in the absence of clinical signs or symptoms. There is no international consensus as to the pattern of follow-up. In France, the recommendations for follow-up are as follows.

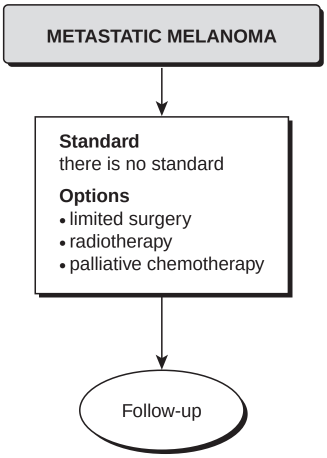

**Figure 3** Treatment of metastatic melonoma

### Melanoma in situ

After complete excision with adequate excision margins, the risk of local recurrence is negligible. Patients should be followed annually throughout life in order to detect a second melanoma.

### Melanoma with a Breslow index of less than 1.5 mm

Follow-up every 6 months for 10 years, then annually throughout life.

© 2001 FNCLCC

### Melanoma with a Breslow index of greater than 1.5 mm or with histological regression whatever the depth, or with a level of Clark equal to IV or V

Follow-up every 3 months for 4 years, every 6 months for years 5–10, then annually throughout life. The rate of recurrence beyond the fifth year is the same whatever the Breslow index of the initial tumour.

### Advanced disease

There is no consensus regarding the approach to follow-up.

### INTERNAL REVIEWERS

MF Avril (Institut Gustave Roussy, Villejuif), C Borel (Centre Paul Strauss, Strasbourg), E Cabarrot (Centre Claudius Régaud, Toulouse), C Carrie (Centre Léon Bérard, Lyon), C Chevreau (Centre Claudius Régaud, Toulouse), C de Gislain (Centre Georges François Leclerc, Dijon), MM Delaunay (Institut Bergonié, Bordeaux), JB Dubois (Centre Val d'Aurelle, Montpellier), MC Escande (Institut Curie, Paris), J Fraisse (Centre Georges-François Leclerc, Dijon), RM Parache (Centre Alexis Vautrin, Nancy), A Ravaud (Institut Bergonié, Bordeaux), H Sancho-Garnier (Centre Val d'Aurelle, Montpellier), and A Spatz (Institut Gustave Roussy, Villejuif).

### EXTERNAL REVIEWERS

P Autier (Institut Jules Bardet, Bruxelles), JM Bonnetblanc (Hôpital Dupuytren, Limoges), S de Raucourt (Hôpital Côte de Nacre, Caen), B Dreno (CHU-Hôtel Dieu, Nantes), MC Gouttebel (Centre Hospitalier, Roanne), F Grange (Hôpital Pasteur, Colmar), B Guillot (Hôpital Caremeau, Nîmes), D Leroy (CHU-Hôpital Clemenceau, Caen), J Revuz (Hôpital Henri Mondor, Creteil), P Saiag (CHU, Boulogne-Billancourt), JL Schmutz (Hôpital Maringer, Nancy), F Truchetet (Hôpital de Bon Secours, Metz), R Regal (Clinique Clémentville, Montpellier) and P Vuillemin (Aix-en-Provence).

*© 2001 FNCLCC*

